# Construction of an easily detectable transgenic *Synechococcus elongatus PCC* 7942 against White Spot Syndrome Virus using *vp*28 and *mOrange* Gene and its metabolism in shrimp

**DOI:** 10.3389/fimmu.2022.974014

**Published:** 2022-08-26

**Authors:** Wen Peng, Qunjing Bao, Rui Jia, Peimin He

**Affiliations:** College of Marine Ecology and Environment, Shanghai Ocean University, Shanghai, China

**Keywords:** VP28-mOrange, *Synechococcus elongatus* PCC 7942, Litopenaeus Vannamei, WSSV, enzyme activity

## Abstract

White spot syndrome is an epidemic disease caused by the highly contagious and lethal white spot syndrome virus (WSSV), resulting in huge economic losses to the global aquaculture industry. VP28 is the main structural protein in the capsule of WSSV and is important in the early stage of infection. Under an excitation wavelength of 548 nm, the mOrange fluorescent protein releases a 562 nm emission wavelength, which is different from the autofluorescence of cyanobacteria. Therefore, using this characteristic combined with the receptor system of *Synechococcus elongatus* PCC 7942, we constructed transgenic *S. elongatus* to express the recombinant protein VP28-mOrange. In addition, PCR and western blotting were used to confirm the stable expression of the target gene in cyanobacteria. Using mOrange tracer features, we explored the recombinant protein VP28-mOrange in the metabolic cycle of young *Litopenaeus Vannamei* after feeding. After the young shrimp had stopped consuming transgenic cyanobacteria, the 24 to 33 h fluorescence signal in the intestine was very weak, and almost disappeared after 36 h. We explored the protective effect of transgenic *vp*28-*mOrange S. elongatus* within 48 h of being ingested by *L. vannamei* and set WSSV challenges at 2, 12, 24, and 48 h post-immunization. However, the survival rate of *L. vannamei* decreased as the time of the WSSV challenge increased. The survival rate on the seventh day was 81%, 52%, 45.5%, and 33.3% for shrimps challenged for 2, 12, 24, and 48 h, respectively. Enzyme activity can also support this conjecture, the enzyme activity indexes of the experimental groups were significantly reduced compared to positive and wild-type controls. Therefore, this immune agent functioned as a preventive agent. Compared with the traditional method, this method was easy to detect and can visualize the digestion of transgenic cyanobacteria in the *Litopenaeus vannamei* intestine.

## Introduction

White Spot Syndrome Virus (WSSV), regarded as a potential pathogen, can infect shrimp at various stages and go undetected until it causes significant disease outbreaks (1). WSSV has been extensively studied over the past 30 years, expanding our knowledge of WSSV genomics and proteomics (2). VP28, the envelop protein of WSSV, promotes attachment and fusion to the host cell during infection (3). In recent years, many methods of controlling WSSV based on the VP28 protein have been explored owing to its remarkable capability to localize on the surface of prawn epithelial cells (4). Many studies have reported that the expression of the recombinant VP28 in heterologous hosts can protect prawns against invading pathogens (5–9). Antibodies produced against VP28 can also effectively control WSSV infection. Monoclonal antibodies produced from mouse hybridoma cells target viral antigens to control the spread of WSSV (10–13). Kim et al. showed that the anti-VP28 egg yolk antibody (IgY) can neutralize WSSV and retains a high survival rate after 15 days of infection, with potential use in immunodiagnosis and immunotherapy (14).

As a model organism, cyanobacteria have a simple structure, rapid growth, strong adaptability, easy genetic manipulation, and most do not contain endotoxin, so they can be used as a good genetic engineering receptor system (15). Cyanobacteria contain a unique pigment-protein – phycocyanin. There has been considerable research and exploration on the characteristic fluorescence spectrum of phycocyanin, and its characteristic wavelength is generally at an excitation of 590–620 nm and an emission of 652–660 nm (16–18). Although the broad spectrum of characteristic fluorescence in cyanobacteria is not uniform, restricting the development of biomass detection, the main purpose of this experiment is to select fluorescent proteins (FPs) that can avoid the fluorescence spectrum of cyanobacteria as marker proteins. Ruffing et al. selected mOrange as a marker protein in *Synechococcus* sp. PCC7002 and showed that transgenic and wild-type cyanobacteria could be distinguished on the spectrogram (19). This provides a reference and basis for using mOrange as a marker protein in cyanobacteria. Previous studies have also shown that orange and red FPs are suitable for cytoplasmic expression in *Chlamydomonas reinhardti*i *(* 20). Orange FPs are also used in plants as marker proteins. Mann et al. transferred and expressed six FP genes in tobacco and *Arabidopsis*. Results showed that orange FP can be used in plant transformation (21).

Based on the above studies, the main purpose of this research was to combine VP28 and mOrange to construct easily detected transgenic *vp*28-*mOrange S. elongatus*. The metabolic and protective effects of transgenic cyanobacteria in *L. Vannamei* were observed, and the mechanism of immune effect was discussed.

## Material and methods

### Experimental materials

*S. elongatus* PCC 7942 (Freshwater Algae Culture Collection at the Institute of Hydrobiology, Wuhan, China) and its transgenic counterparts expressing VP28 (*S. elongatus* harboring pRL-489-vp28, Institute of Botany, Chinese Academy of Sciences, Shanghai, China) grown in BG-11 medium at 30°C with 120 rpm shaking and continuous light (50 μmol photons m^−2^ s^−1^) in BG-11 medium (22). E. coli was grown in Luria-Bertani medium at 37°C. Dr. D.J. Shi provided the pRL-489 in E. coli DH5 and the RP4 + pRL-542 in E. coli.

The post-larvae of *L. vannamei* were obtained from Zhongzheng Aquatic Products Co., Ltd., Hainan, China. The culture conditions were filtered in circulating water with a salinity of 16‰, the water temperature was maintained at 28–30°C, and the shrimp were fed twice a day with Artemia cysts at 5% of their body weight in a 200 L aerated circular aquarium. After 2 months of culture, the average body length grew from 1 cm to 3–4 cm (**Figure S1**).

WSSV was obtained from the Yellow Sea Fisheries Research Institute, Chinese Academy of Fisheries Sciences, Shanghai, China, for the viral challenge in *L. vannamei. Macrobrachium nipponensis* (Shanghai Guoyuan, China) was used to rejuvenate the virus, moribund individuals were collected, and prawn muscle was cut into small pellets and stored at -80°C (23).

### Experimental methods

The *vp*28 and *mOrange* genes were cloned, shuttle expression vectors were constructed, and *S. elongatus* was transformed. The accession numbers of the *vp*28 and *mOrange* genes are GenBank ABG74923.1 and AAV52165.1, respectively.

The two fragments were amplified by PCR and fused with linker primer (GGGGS) using overlap PCR. The fusion fragment was digested with *Xho*I and *Kpn*I for the integration of pRL-489 between the same restriction sites, then transferred into the *E. coli* Top10. The shuttle expression vector includes antibiotic resistance (KmR), the promoter PpsbA and terminator regions flank the fusion fragment. Primers were used to construct these fragments and the sequences of the constructing shuttle expression vectors can be found in the Additional file and **Table S1**.

*S. elongatus* was transformed by tri-parental conjugative transfer (24). Typically, the *E. coli* Top10 harboring the shuttle expression plasmid and the conjugal and helper plasmid RP4 + pRL542 were harvested and mixed with fresh cyanobacterial cells. The mixtures of *E. coli* and cyanobacteria were screened for transgenic colonies on BG-11 plates with kanamycin sulfate. PCR was used to detect whether the target gene existed in the transgenic *vp*28-*mOrange S. elongatus*. PCR conditions and primers can be found in the supplementary file (**Table S2**).

### Western blot analysis

During the late logarithmic growth phase, the wild-type and two *S. elongatus* of *vp*28-*mOrange* were collected. Their soluble proteins were extracted at -20°C using a freeze-thaw method, and equally loaded proteins (10 g) were separated using 12 percent sodium dodecyl sulfate polyacrylamide gel electrophoresis (SDS-PAGE) before being blotted onto a polyvinylidene difluoride membrane (Merck Millipore, Massachusetts, USA). For 1.5 hours, the membrane was blocked with 5% (w/v) nonfat milk in 0.05 percent (w/v) Tween 20, 10 mM Tris (pH 8.0), and 150 mM NaCl. After adding the VP28 and mOrange monoclonal antibodies (1:5,000) from Abmart Inc., the membrane was incubated at room temperature for 1.5 hours. Following washing, a goat anti-rabbit IgG Horseradish Peroxidase (HRP) conjugated secondary antibody (1:5,000) (Bio-Rad, Hercules, USA) was added and incubated for 1.5 hours at room temperature. Protein gel blot analysis was carried out using an ECL Plus assay Kit (Shangon, Shanghai, China) in accordance with the manufacturer’s instructions (22).

### Measurement of fluorescent protein spectra

Cyanobacterial colonies and cells of wild-type and *vp*28-*mOrange* mutant of *S. elongatus* were observed under a fluorescence microscope (Nikon Eclipse NI-U, Japan) using mOrange filters (ET530/30x; ET575/40m). The cyanobacterial colonies on a solid medium were observed under a 4-fold objective lens directly. Cells in liquid medium were dropped onto a glass slide and observed under a 20-fold objective lens directly. Exposure times were 350 ms and 1500 ms.

### Real-time quantitative polymerase chain reaction was used to measure *vp28* gene expression

The cyanobacterial stock solution was added to fresh BG-11 medium (150 ml) according to the inoculation ratio of 1:100, and the culture was started. On the 3rd, 6th, 9th, 12th, and 15th day of culture, the algae were collected to measure the expression level of *vp*28-*mOrange* gene. Total RNA from the cells of mutant *S. elongatus* collected at different growth times (3, 6, 9, 12, and 15 days) were extracted and reverse-transcribed into cDNA using a FastQuant RT kit (Tiangen, Beijing, China). Primers (see **Table S3**) designed according to *vp*28-*mOrange* genes were used to quantify gene expression. The *Sec*A gene was used as an internal reference gene to normalize the data. The reaction mixture was prepared using SYBR Green real-time PCR Master Mix (Tiangen). Final concentrations in a total volume of 25 μL were as follows: 12.5 μL of ×2 SuperRealPreMix Plus (including SYBR Green I), 0.75 μL of 10 mM forward and reverse primers, and 2 μL of cDNA, 0.5 μL×50 reference dye, and 8.5 μL RNase-free ddH_2_O. The 2^−△△Ct^ method was used to assess the relative quantification of gene expression (25). Using an RT-qPCR system, the following PCR protocol was used: denaturation at 95°C for 15 min, 40 cycles at 95°C for 10 s, followed by 55°C for 20 s, and extension at 72°C for 30 s using an RT-qPCR system (Funglyn FTC-3000, Toronto, Canada).

### Immunization experiment

Wild-type and *S. elongatus* mutants harboring *vp*28 and *vp*28-*mOrange* were cultivated at 30°C with shaking at 120 rpm for 12 days and then collected by centrifugation. Pellets were washed twice with fresh BG-11 medium, then freeze-dried using a freeze dryer. At least 0.5–0.6 g of dried cyanobacterial powder could be harvested from 1 L of *S. elongatus* solution.

*L. vannamei* were divided at random into eight experimental groups: positive control group (challenge and no immunization), negative control group (neither immunization nor challenge), wild-type group (challenged and fed with *S. elongatus*), vp28 group (challenged and fed with *S. elongatus* harboring *vp*28), and v+m-7942-C2/C12/C24/C48 groups (challenged at 2, 12, 24, and 48 hours after fed with *S. elongatus* harboring *vp*28-*mOrange*), and each trial was conducted in triplicate (30 prawns/group/replication).

The prawns of each group were immunized for 10 days by feeding with Artemia cysts mixed with cyanobacteria powder three times every day at 5% body weight and a protein dose of 10 μg/tail. The v+m-7942-C2/C12/C24/C48 groups were fed *vp*28-*mOrange S. elongatus*, wild type group was fed wild type *S. elongatus*, vp28-7942 group was fed *vp*28 *S. elongatus*, positive control and negative control were fed ordinary food. Observe that after the food blocks in the shrimp intestines are completely excreted, *L. vannamei* were challenged by feeding with the muscles of WSSV-infected by *M. nipponensi*. The v+m-7942-C2/C12/C24/C48 groups were challenged at 2, 12, 24, and 48 hours after the final immunization, the vp28-7942 group,wild type group and positive control were challenged at 2 hours after the immunization. After 4 h, prawns were washed and transferred to WSSV-free seawater in a new culture tank. Cumulative mortality was recorded in each group from the start of the challenge, dead prawns were collected every day, and PCR was performed to test for WSSV infection.

### Distribution and metabolic cycle of *vp28-mOrange* fusion protein *in vivo*

Ten shrimps (around 1 cm) were selected and fed with *vp*28-*mOrange S. elongatus* 7942 for 24h after starvation treatment. After the intestinal mucosa was observed to be full of green algae, the young shrimps were transferred to clean water for temporary rearing. The distribution of fluorescence signal and the duration of fluorescence signal in shrimps were observed and photographed by mOrange filter group under 4x objective lens every 3h, and the young shrimps fed ordinary diet were used as control. Image-pro Plus 6.0 was used for grayscale analysis of fluorescence images of juvenile shrimp midgut at different time points, and the average optical density value was calculated according to the following formula after obtaining corresponding parameters:


(4–1) $$\text{density }\left(\text{mean}\right)=\frac{\text{intergated optical density}\left(\text{IOD}\right)}{\text{area}}$$

The average optical density value can reflect the intensity of light signal in the image. In this study, the intensity of fluorescence signal of mOrange fluorescent protein in the midgut of juvenile shrimp was used to reflect the metabolism of VP28-mOrange, the recombinant protein expressed by cyanobacteria, in the midgut of juvenile shrimp after feeding the transgenic cyanobacteria, and the average value was calculated to provide a basis for the subsequent challenge time. 

### Enzyme activity detection

At 2, 12, 24, and 48 hours after the challenge, the muscles of five prawns were randomly collected from the positive group, negative group, v+m-7942-C2group (v+m-7942), vp28-7942group, and wild-type group (about 0.1 g maintained at 4°C on ice) and stored at -80°C. The samples were homogenized in a 1:9 ratio with precooled 0.86 percent physiological saline and centrifuged for 15 minutes (2,000 rpm at 4°C). The precipitate was discarded, but the supernatant was kept to measure changes in superoxide dismutase (SOD), catalase (CAT), prophenoloxidase (PO), and fatty acid synthase (FAS) activity using an Enzyme Activity Assay Kit (Jiancheng, Ltd., Nanjing, China) and read on a Microplate spectrophotometer (i Mark, Bio-Rad),.

## Results

### Construction of transgenic *vp*28-*mOrange S. elongatus*

**Figure 1:**
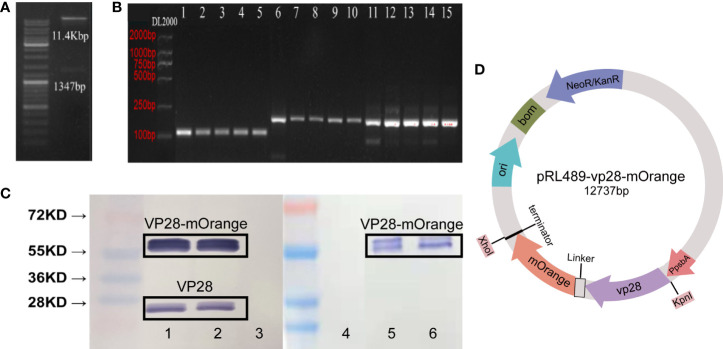
**(A)** Identification of expression vectors pRL-vp28+mOrange plasmid by enzyme digestion; **(B)** PCR product electrophoresis verification. The template used in Line1 and 6 is a plasmid as a positive control. The template used in Line11 is wild-type, and the other line templates are vp28-mOrange genotype, with four biological replicates. Line 1-5: vp28-1 primer + cDNA template; line 6-10: mOrange-1 primers + cDNA template; line 11-15: 16s RNA primers + cDNA template; **(C)** Western blot analysis of VP28-mOrange fusion protein from transgenic *S. elongatus*. Band 1, 2, 5, 6: vp28-mOrange genotype, band 3, 4: wild-type. Band 1-3: VP28 antibody, band 4-6: mOrange antibody. Lane 1 and 2 are repeated experiments in VP28 antibody group, lane 3 and 4 are repeated experiments in mOrange antibody group. Bands around 55kDa in lane 1 and lane 2 are VP28-mOrange fusion proteins, while bands around 28kDa are VP28 proteins; **(D)** Plasmid structure of pRL489-vp28-mOrange.

The *vp*28-*mOrange* gene fragment was first amplified and confirmed by sequencing using splicing overlap extension (SOE) PCR. The sequence identity between GenBank’s *vp*28-*mOrange* and the cloned fragment was 100 percent. Second, a vector containing the *vp*28-*mOrange* gene was created (**Figure 1D**) and confirmed by restriction endonuclease digestion (**Figure 1A**). Third, total RNA was extracted from transgenic cyanobacteria and reverse-transcribed into cDNA. For PCR amplification and confirmation, *vp*28 and *mOrange* primers were used. The PCR products formed using the two groups of primers produced single bands, as shown in **Figure 1B**, which were consistent with the expected product sizes.

Sequence comparison and monoclonal antibody were used to detect VP28 protein. The soluble protein was extracted from wild-type and transgenic *vp*28-*mOrange S. elongatus* cells cultured to logarithmic phase, and the protein concentration of the algae was measured by the Bradford method to determine the loading amount of protein electrophoresis. Two antibodies (VP28, mOrange) were used to detect the same fusion protein. The fusion protein was detected with VP28 antibodies and the results showed bands 1, 2 and 3. Bands 4, 5 and 6 the used mOrange antibody (**Figure 1C**). Western blot results showed that the shuttle expression vector has been transformed and expressed recombinant protein in *S. elongatus*. The molecular weight of the protein was around 55 KD. In the response of vp28 antibody group, bands 1-2 with sizes between 20 and 28K appeared. Through analysis, we speculate that these bands are vp28 protein. We speculated that the reason for this strip was that the terminator of *vp*28 gene sequence was not deleted in pRL-489-vp28-mOrange plasmid, which is likely to result in the expression of a single *vp*28 gene and thus recognized by VP28 antibody.

### Microscopy of fluorescent protein reporters for gene expression

Fluorescent protein reporters are often used in chassis organisms to analyze gene expression. The use of FP reporters in cyanobacteria is complicated by the absorbance and fluorescence of native photosynthetic pigments. Therefore, FP reporters for *S. elongatus* were based on the absorbance and fluorescence spectra for this host.

**Figure 2:**
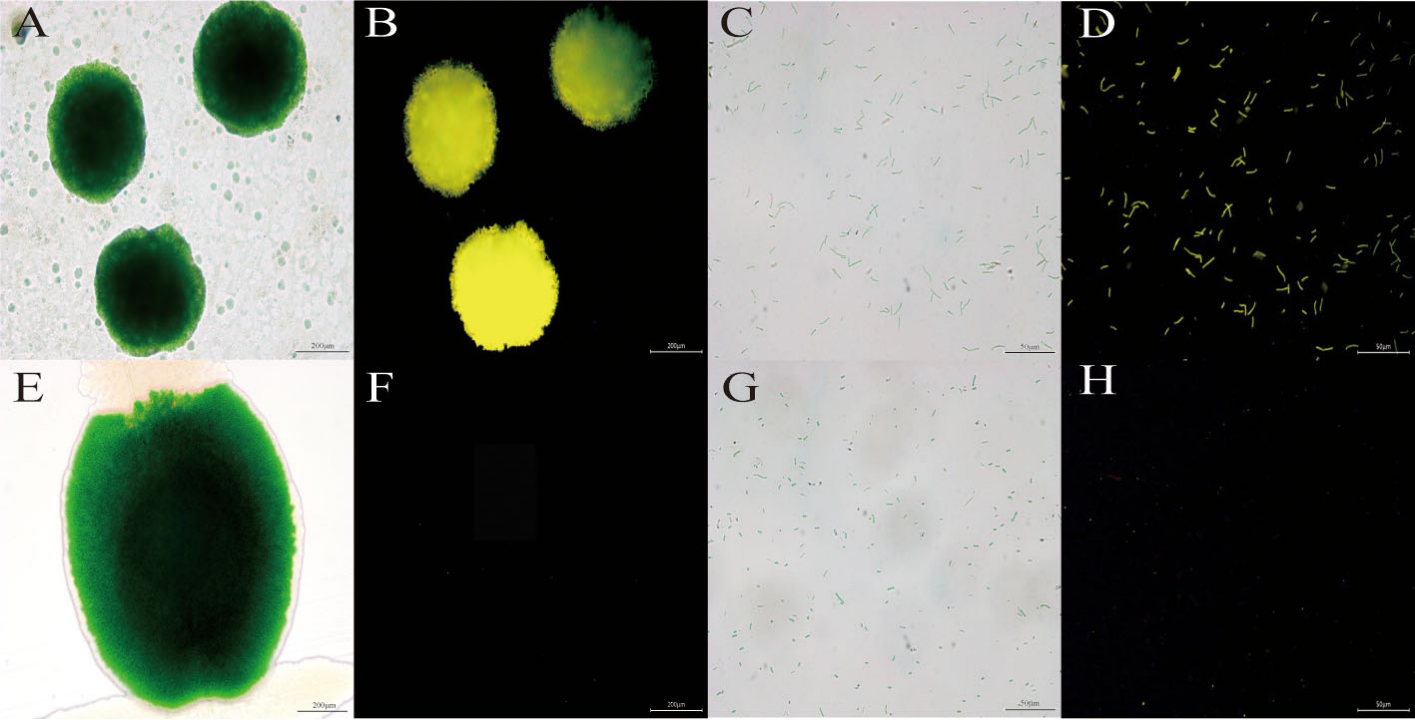
The image of cyanobacteria colonies and cells under fluorescence microscopy. **(A, B)** Transgenic vp28-mOrange S. elongatus in light field and mOrange filter. **(E, F)** Wild S. elongatus in light field and mOrange filter. **(C, D)** Transgenic vp28-mOrange S. elongatus in light field and mOrange filter. **(G, H)** Wild S. elongatus in light field and mOrange filter.

As can be seen from **Figure 2**, both the algal colonies (D) and algal cells (B) transformed into transgenic *vp*28-*mOrange S. elongatus* emitted bright fluorescence under the fluorescence field, while the wild-type (F, H) only showed faint red spontaneous fluorescence under the fluorescence field. It was found that the FP in the VP28-mOrange fusion protein exhibited the fluorescence properties normally, and there was a significant difference between the wild-type and transgenic cyanobacteria in the fluorescence field, which made it easy to distinguish the two and thus achieved the purpose of easy detection.

### The quantification of *vp*28-*mOrange* gene expression in transgenic mutants

**Figure 3:**
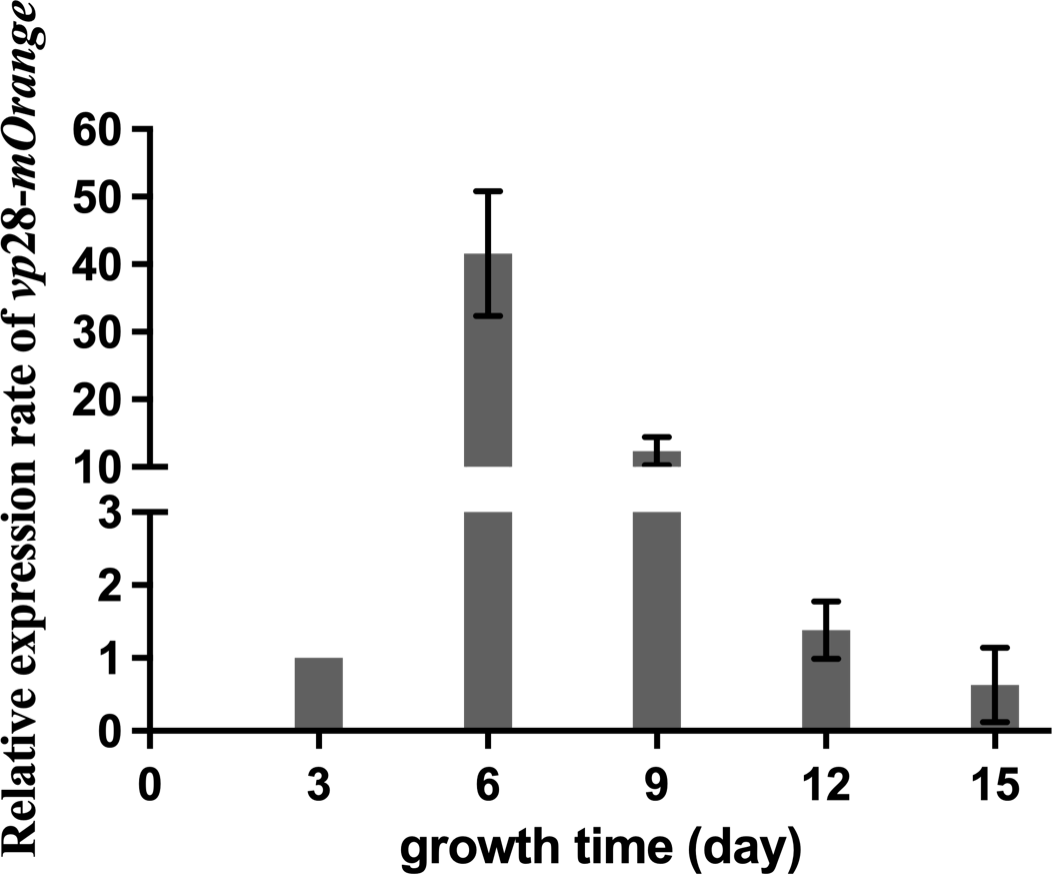
*vp*28-*mOrange* gene expression of mutant *S. elongatus* on the 3rd, 6th, 9th, 12th and 15th days of growth.

We collected the transgenic mutants during growth and calculated *vp*28-*mOrange* expression (**Figure 3**). The expression level of the *vp*28-*mOrange* gene was measured and normalized to the third day. The results showed that the relative expression level of the *vp*28-*mOrange* gene was the highest on the sixth day, which was 41.61 times the expression level on the third day, and then the expression level decreased gradually. Amplification and melting curves indicated that the primers were amplified normally and had no stray bands (**Figure S2**).

### Immunization experiment

**Figure 4:**
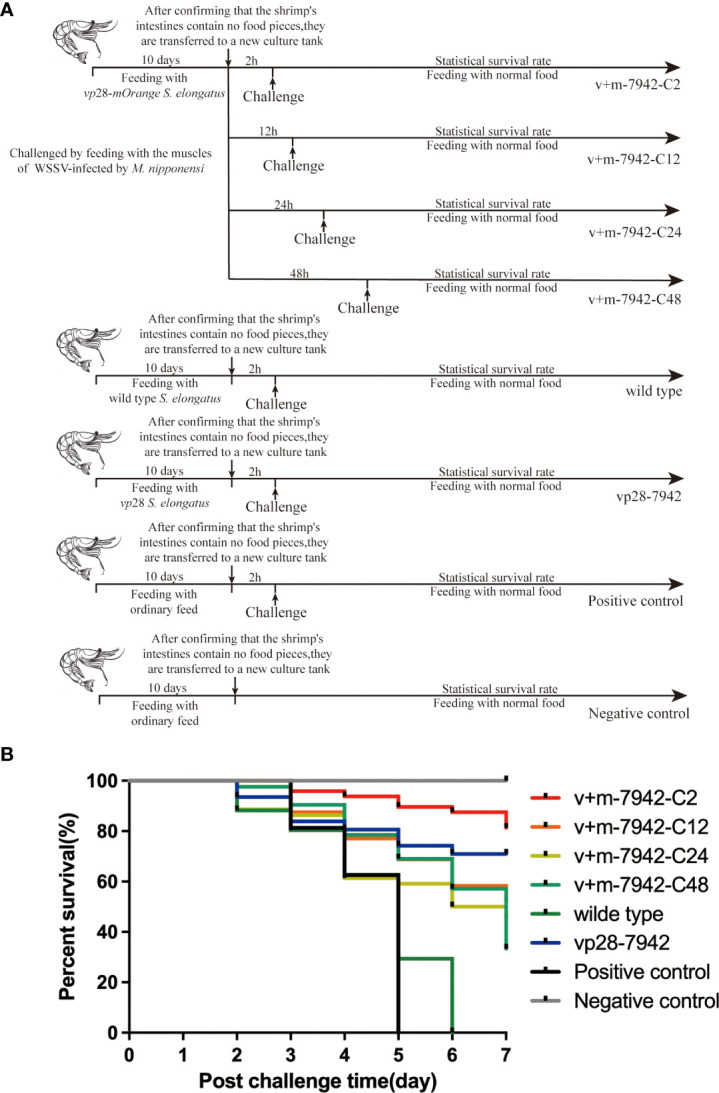
**(A)** *L. vannamei* survival protocol: positive control group (challenge and no immunization), negative control group (neither immunization nor challenge), wild-type group (challenged and fed with *S. elongatus*), vp28 group (challenged and fed with *S. elongatus* harboring *vp*28), and v+m-7942-C2/C12/C24/C48 groups (challenged at 2, 12, 24, and 48 hours after fed with *S. elongatus* harboring *vp*28-*mOrange*), and each trial was conducted in triplicate (30 prawns/group/replication); **(B)** Cumulative survival of *L. vannamei* after WSSV infection. .

According to our research (**Figure 4**), the death rate of the positive control group reached 100% on day 5, while the negative control group maintained a 100% survival rate. In contrast, the mortality rate in the wild-type control group increased rapidly within 2–5 days, and all died on the sixth day, proving that wild-type *S. elongatus* could not play a protective role. However, on the seventh day, the survival rate of the *vp*28-7942 group reached 71% and the survival rate of v+m-7942-C2 in the experimental group reached 81%, which proved that mOrange did not affect the efficacy of VP28 in the recombinant protein molecules. The protection efficiency was even higher than that of the transgenic *vp*28 type *S. elongatus*. The recombinant protein VP28-mOrange had no toxicity, meanwhile, it had a good protective effect on *L. vannamei.*

The survival rates of experimental groups v+m-7942-C2, v+m-7942-C12, v+m-7942-C24, and v+m-7942-C48 decreased sequentially on the seventh day, which was 81%, 52%, 45.5%, and 33.3%, respectively. The protection effect was the best at 2 h and the protection effect is the worst at 48 h. This proved that the protective effect of transgenic *vp*28-*mOrange S. elongatus* decreased with time but still had a certain protective effect after 48 h. At 2 h, the protection offered by transgenic *vp*28-*mOrange S. elongatus* was better than that of transgenic *vp*28 *S. elongatus.*

### Localization and the metabolic cycle of *vp28-mOrange* fusion protein *in vivo*

**Figure 5:**
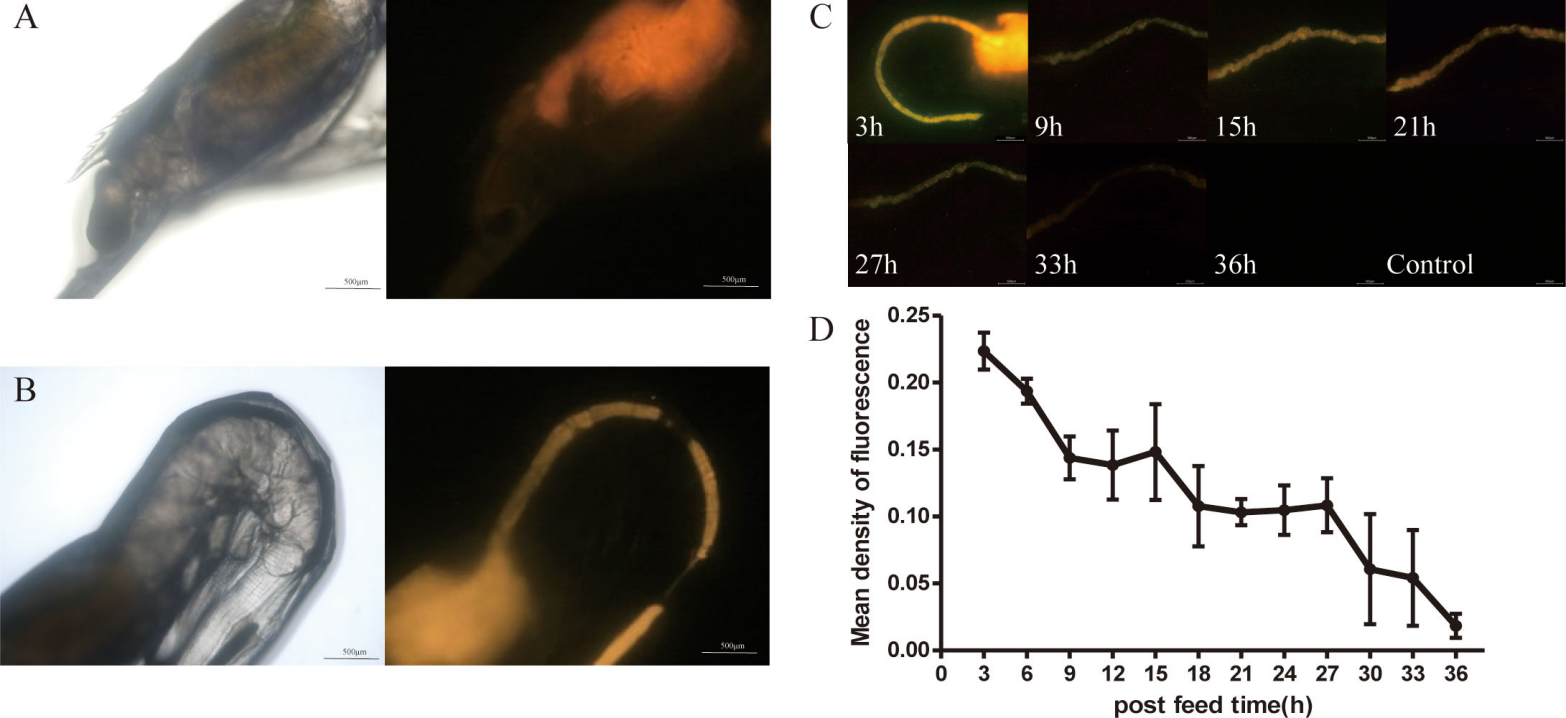
Metabolism of VP28-mOrange fusion protein in *L. vannamei*. Distribution of VP28-mOrange fusion protein in *L. vannamei* stomach **(A)**, midgut **(B)**; **(C)** The retention time of VP28-mOrange fusion protein in prawn gut; **(D)** Mean density of fluorescence in the midgut of prawn.

Fluorescence microscope images (**Figures 5A, B**, **Figure S3**) showed that when transgenic *vp*28-*mOrange S. elongatus* was fed to prawns, the fluorescence signal was mainly distributed in the stomach, gills, and midgut and a small amount was distributed in the eyes.

First, the metabolic status of the *S. elongatus* containing the fusion protein VP28-mOrange in the midgut after being ingested by juvenile shrimp was observed by a fluorescence microscope (**Figure 5C**). The results showed that the fluorescence signal in the intestinal tract of juvenile shrimp was gradually weakened after feeding the transgenic cyanobacteria was stopped. The fluorescence signal was weakened after 24–33 h and gradually disappeared after 33 h (not shown).

The results have indicated that the recombinant protein VP28-mOrange in transgenic cyanobacteria was gradually metabolized and degraded *in vivo* or excreted *in vitro*. The average fluorescence density in the midgut of the prawn at 33 h decreased by 3.5 times compared with 3 h, and the recombinant protein could stay in the midgut of the prawn for about 33 h (**Figure 5D**).

### Enzyme activity results

**Figure 6:**
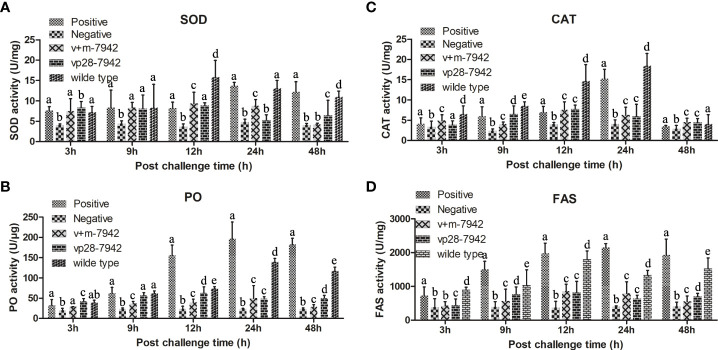
Different enzyme activities were detected after the WSSV challenge. The same lower case letters in the figure indicate that the difference is not significant (P > 0.05), and different lowercase letters indicate a significant difference (P < 0.05). The activity of **(A)** SOD; **(B)** PO; **(C)** CAT; and **(D)** FAS in different groups of *L. vannamei* after infection. .

In this study, the activities of four enzymes were detected, namely SOD, CAT, PO, and FAS (**Figure 6**). Except for the negative control group, the activities of the four enzymes in each group first increased and then decreased with the prolongation of infection time. Between 0 and 12 h, SOD, CAT, and FAS in the wild-type groups increased significantly and the activities of FAS and PO in the positive control groups increased significantly. Between 12 and 24 h, the activity of SOD, CAT, and FAS in the positive control group was equal to the wild-type group and significantly higher than the other groups. PO in the positive control group was higher than in the wild-type. The overall activities of SOD, CAT, FAS, and PO in the v+m-7942 group and vp28-7942 groups were lower than those in the positive control and wild-type groups, and the peak was at 12–24 hours. There was no significant difference in the activities of the four enzymes at each time point in the negative control group (P>0.05).

## Discussion

### Constructed transgenic *vp*28-*mOrange S. elongatus* and easy detection

DNA vaccines (plasmids encoding the viral protein VP28) provide a unique approach to WSSV control. Numerous studies have shown that plasmid DNA vaccination can confer resistance to WSSV in prawns (26, 27). Therefore, *vp28* was selected as the target gene in this research. In recent years, the progress of research is rapid, and it has been widely used as a marker protein in the fields of biology and medicine (28). Cyanobacteria themselves carry a large quantity of photosynthetic pigments. The marker proteins are essential for the fluorescent labeling of cyanobacteria. The emission wavelengths of orange FP and cyanobacterial autofluorescent protein are different, which is an important reason for the selection of orange FP mOrange. This study successfully constructed a plasmid vector containing *vp28* and *mOrange* genes (**Figure 1D**). It is worth mentioning that when designing sequences, flexible linking peptides (GGGGS) were added between the two short proteins (29) because glycine with a low molecular weight can reduce the conformation restriction on the peptide skeleton. The added peptide chain can obtain sufficient space for folding, thus ensuring the original activity and improving the success rate of fusion expression. PCR and western blot analysis showed that we successfully introduced a recombinant plasmid capable of expressing the *vp*28-*mOrange* gene into *S. elongatus*, and constructed the transgenic *vp*28-*mOrange S. elongatus* PCC 7942 strain. The 55 kDa band in **Figure 1C** represents the fusion protein. The band in 28kDa is the VP28 protein band. This band appears because the terminator of the *vp*28 gene sequence located upstream is not deleted in the plasmid, so in the process of prokaryotic transcription and expression, ribosomes are constantly on the mRNA chain. Going forward, when it recognizes the terminator of the vp28 sequence, it will terminate the translation and detach from the mRNA chain, resulting in the expression of a separate VP28 protein. Since the translation process of prokaryotes is not so precise and strict, *vp*28 and the *mOrange* gene sequence is close, so the complete fusion protein will still be expressed. The recombinant plasmid pRL-vp28+mOrange was stably inherited in *S. elongatus*, the fusion expression of the exogenous gene *vp*28 and *mOrange* was successful, and the image of the *E. coli* containing the recombinant plasmid under the fluorescent field showed that mOrange still had fluorescence activity in the recombinant protein expressed by fusion. This has laid the foundation for the subsequent identification of transgenic cyanobacteria by fluorescence signal and the quantification of the expression efficiency of exogenous genes in *S. elongatus* PCC 7942 by fluorescence intensity.

As a model organism, cyanobacteria have a simple structure, rapid growth, and strong adaptability. In addition, autotrophic photosynthetic organisms can synthesize organic matter from inorganic substances, the expressed proteins cannot easily form inclusion bodies, and can be used as a good genetic engineering receptor system (15). In previous studies, the *vp*28 gene has been successfully expressed in *Anabaena* sp. PCC 7120 to produce a subunit vaccine with a protection efficiency of 68.0% (22). In factory production, our cyanobacteria must be easily detectable, while remaining effective. Using PCR or western blotting is not only time-consuming but also expensive. FP is a more commonly used labeling method, but it is rarely used in cyanobacteria. Because of the strong fluorescence background with light, pigment, and phycocyanin, it is necessary to select an FP that is suitable for the fluorescence characteristics of cyanobacteria. The mOrange protein releases an emission light at 562 nm under an excitation wavelength of 548 nm, which is an emission spectral characteristic that is distinct from cyanobacterial autofluorescence and has been used as a marker protein in *Synechococcus* sp. PCC 7002 (19). Fluorescence microscopy images (**Figure 2**) showed that the transgenic *vp*28-*mOrange S. elongatus* has a bright orange-yellow fluorescence signal, while the wild-type did not show an orange fluorescent signal under the same exposure conditions. According to this, the transgenic and wild-type *S. elongatus* can be easily distinguished by fluorescence microscopy and corresponding filter sets. It is conceivable that if this technology is used in the production process, the identification of transgenic cyanobacteria can be completed within 2–3 minutes, which greatly shortens the quality inspection time. The results of qPCR showed that the RNA transcription level of the target gene reached a peak on the sixth day, which was 41.61 times that of the third day and then decreased gradually. The reason for the rapid decline of vp28 expression after 6 days may be that the transcription and expression of genes will be inhibited through a negative feedback regulation mechanism after the accumulation of exogenous proteins in cells reaches a certain level. This result can guide the production process. It is recommended that the harvesting time of industrial production of transgenic cyanobacteria should be carried out within 6-12 days. At this time, the density of cyanobacteria does not reach the highest level, the metabolic waste in the medium is low, and the level of nutrient consumption is relatively low, which can be recycled.

### The pesticide effect of transgenic *vp*28-*mOrange S. elongatus*

Yi et al. have demonstrated that VP28 had a strong specific binding ability to prawn cells and could enter the cytoplasm after adsorption for 3 h. It was inferred that WSSV may recognize and bind to a receptor on the host cell membrane mediated by VP28, and then the virus was endocytosed by the host cell and finally released into the cytoplasm (3). Subsequent research results continue to support this inference (30). Kulkarni et al. used immunome methods to confirm that the VP28 recombinant protein, inactivated WSSV, and live WSSV could adhere to the borders of the midgut epithelial cells in *Penaeus monodon* within 2 h, and initiate receptor-mediated endocytosis, endocytic vesicles aggregate and bind to lysosomes to form supra-nuclear structures (31). Chen et al. found that the envelope protein VP28 can bind to γ-aminobutyric acid receptor-associated protein (GABARAP), an important member of the Atg8 family on the cell membrane of crayfish hematopoietic cells, and the binding complex can bind to cytoskeletal proteins. The WSSV envelope protein VP28 can interact with proteins on the host cell membrane at the early stage of infection to initiate virus invasion (32). And adding additional VP28 protein in the experiment will compete with VP28 on the virion for binding to Cq-GABARAP protein to reduce virus entry. Transgenic *vp*28-*mOrange S. elongatus* can successfully express VP28 protein(**Figure 1C**). From this, it can be inferred that in previous studies (3, 30), recombinant VP28 protein was orally entered into the host intestine, where it bound to relevant proteins on the surface of host cells, and preempted the relevant sites that were originally capable of binding to WSSV. Thus, the binding of the later invading WSSV to the host cell is competitively inhibited, so that the host cell avoids or reduces the probability of being invaded by WSSV, thereby protecting the host. From this, it can be inferred that in previous studies (3, 30), recombinant VP28 protein was orally entered into the host intestine, where it bound to relevant proteins on the surface of host cells, and preempted the relevant sites that were originally capable of binding to WSSV. Thus, the binding of the later invading WSSV to the host cell is competitively inhibited, so that the host cell avoids or reduces the probability of being invaded by WSSV, thereby protecting the host. In this research, using the tracer properties of VP28-mOrange, direct observation by fluorescence microscopy, and image analysis software Image-Pro Plus 6.0, the metabolic cycle of *S. elongatus* containing recombinant protein VP28-mOrange after being ingested by juveniles was explored. The results showed that after the feeding of transgenic cyanobacteria was stopped, there was a strong fluorescent signal in the stomach, midgut and eyes of the shrimp. Fluorescence in stomach and midgut represents the entry of fusion proteins into the intestine, which may produce competitive immunity. It is speculated that the weak signal in the eyes may be due to the presence of some membrane proteins that can bind VP28 protein on the surface of the epithelial cells in the eyes of shrimp, and the tiny structure of the eyes of shrimp may make the VP28-mOrange fusion protein adsorb on the surface and reflect fluorescence. We think it’s more likely that the shrimp’s eyeball itself will reflect light from the machine above and be caught by the machine. The fluorescence signal in the intestinal tract of juvenile prawns gradually weakened, and the fluorescence signal was very weak at 24–33 h, and basically disappeared at 36 h. Shrimp gut at 9 h in fluorescent darker because before 9 h is shrimp eat algae through the intestinal peristalsis has been part of drain out or has been destroyed by digestive juice fluorescent protein structure, and in this shrimp without eating a new food so led to the decrease of the fluorescence intensity, and in the later, was the food in the stomach and along the gastrointestinal peristalsis to long in the middle, So the fluorescence intensity gets stronger again. Most decapod crustaceans including non-prawns can digest food in 6–12 hours, while it can take up to 24 h to empty the gut completely (33–35). It should be noted that this is the time that food is metabolized, and VP28 recombinant protein can mediate endocytosis into cells and bypass digestion, so in the report by Kulkarni et al., even at 72 h, the VP28 recombinant protein could be detected in the distal midgut lumen (31). In this research, the longest fluorescence signal of juvenile shrimp was 33 h. A study by Kulkarni et al. selected shrimp weighing between 18 and 20 g. We selected a prawn with a small body length, which was convenient for us to observe under the microscope. Food retention time and gut motility, determine the digestive efficiency and assimilation in shrimps (36). Therefore, the residence time of the fluorescence signal was different. On the other hand, it may also be limited to the exogenous genes of cyanobacteria. The low expression efficiency and the low food intake of juvenile shrimp resulted in insufficient intake of recombinant protein VP28-mOrange, which reduced the intensity of the fluorescent signal.

Based on the research results of the metabolic cycle after feeding juvenile shrimp with transgenic *vp*28-*mOrange S. elongatus* and considering that it is impossible to predict when WSSV will break out in the actual application process, we designed and explored a transgenic *vp*28-*mOrange S. elongatus* immunization agent.

### Protective effects of different challenge times within 48 hours after being ingested by prawns

Different post-immunization challenge times (2, 12, 24, and 48 h) were set. The experimental results showed that with the delay in the challenge time after immunization, the survival rate of 81%, 52%, 45.5%, and 33.3% in the 2, 12, 24, and 48 h groups, respectively, decreased sequentially on the seventh day. It was found that the protective effect of transgenic cyanobacteria was the best at 2 h after stopping immunization, and protection was the weakest at 48 h. At the same time, it shows that our fusion protein can effectively prevent WSSV from infecting prawns and improve the survival rate of prawns.

SOD is an enzyme involved in the conversion of superoxide anion to hydrogen peroxide and is the first line of defense when reactive oxygen species (ROS) homeostasis is disturbed (37). CAT is also an important antioxidant defense enzyme in living organisms (38). It can catalyze SOD to generate water and oxygen in the process of scavenging ROS, thereby protecting the stability of the intracellular environment and protecting cells from oxidative damage by ROS (39). PO is an oxidoreductase that exists in crustaceans in the form of prophenol oxidase. It is a key enzyme in melanin synthesis and stimulates multiple cellular defense responses, including phagocytosis, nodule formation, encapsulation, and hemocyte locomotion (40). These three enzymes are the main enzymes that help the body resist foreign pathogens. The results showed that the enzyme activity indicators in the wild-type and positive control groups are significantly higher than those in the experimental group, indicating that the VP28 in the transgenic cyanobacteria fed in the experimental group. The recombinant protein protects the host cells from or delays the invasion of WSSV, and the body itself does not have a strong immune response, so the corresponding enzyme activity indicators in the experimental group are much lower. However, FAS plays a role in the packaging stage of the virus, and long-chain fatty acids assemble the viral envelope through FAS (41). This process generally occurs 24 hours after WSSV invasion. The FAS enzyme activity of the wild-type and the positive control groups was significantly higher than that of the experimental group at 24-48 h, indicating that the WSSV packaging was more frequent in the host cells of the positive control group, and that the content of WSSV in the host cells of the experimental group was lower.

The results of the drug efficacy test showed that after ingesting the transgenic cyanobacteria mixed diet, *L. vannamei* had good resistance to WSSV, and the survival rate was greatly improved (81%) compared with the control group (0%). The recombinant protein expressed by the fusion of mOrange and VP28, mOrange did not reduce the protective effect of VP28 on *L. vannamei*, and even the protection efficiency was higher than that of transgenic *vp*28 *S. elongatus*. The recombinant protein was nontoxic and had a good protective effect, which confirmed that the transgenic cyanobacteria constructed in this study could improve the antiviral ability of prawns.

## Data availability statement

The original contributions presented in the study are included in the article/**Supplementary Material**, further inquiries can be directed to the corresponding authors.

## Author contributions

WP designed the experiments and performed the experiments, data analysis and manuscript writing. QB performed the experiments, analyzes data and writes manuscripts. PH assisted in manuscript editing. RJ designed the experiment and revised the manuscript. All authors contributed to the article and approved the submitted version.

## Funding

This research was supported by the National Key Research and Development Plan (2019YFC0312604), the Shanghai Agriculture Science and Technology Innovation Project (2017, No1–13), the Shanghai Science and Technology Commission Innovation Project (Nos.16391903500 and 17391902200), and the National Marine 863 Project (No.2014AA093506).

## Acknowledgments

We thank International Science Editing (http://www.internationalscienceediting.com) for editing this manuscript.

## Conflict of interest

The authors declare that the research was conducted in the absence of any commercial or financial relationships that could be construed as a potential conflict of interest.

## Publisher’s note

All claims expressed in this article are solely those of the authors and do not necessarily represent those of their affiliated organizations, or those of the publisher, the editors and the reviewers. Any product that may be evaluated in this article, or claim that may be made by its manufacturer, is not guaranteed or endorsed by the publisher.
