# Supplementary material for: Construction of an easily detectable transgenic Synechococcus elongatus PCC 7942 against White Spot Syndrome Virus using vp28 and mOrange Gene and its metabolism in shrimp
Source: Front Immunol. 2022 Aug 26;13:974014. doi: 10.3389/fimmu.2022.974014 (PMC9459150; doi:10.3389/fimmu.2022.974014)
Supplement: Supplementary file 1 [file DataSheet_1.docx]

Supplementary Material

# PCR experiment process

Total RNA from the cells of mutant *S. elongatus* was extracted with RNA extraction kit (Tiangen, China), and reverse transcribed into cDNA with iScript™ gDNA Clear cDNA Synthesis Kit. Afterwards, PCR experiments were performed. The PCR reaction system was 25 μl, 12.5 μl 2×Taq PCR Mastermix, 1 μl forward primer (10 μM), 1 μl reverse primer (10 μM), 1 μL cDNA, and 9.5 μL RNase free dH_2_O.

PCR reaction steps: denaturation at 95 °C for 15 min, 40 cycles at 95 °C for 10 s, followed by 50-60 °C for 20 s, and extension at 72 °C for 30 s. PCR products were detected by electrophoresis.

# Supplementary Figures and Tables

## Supplementary Figures


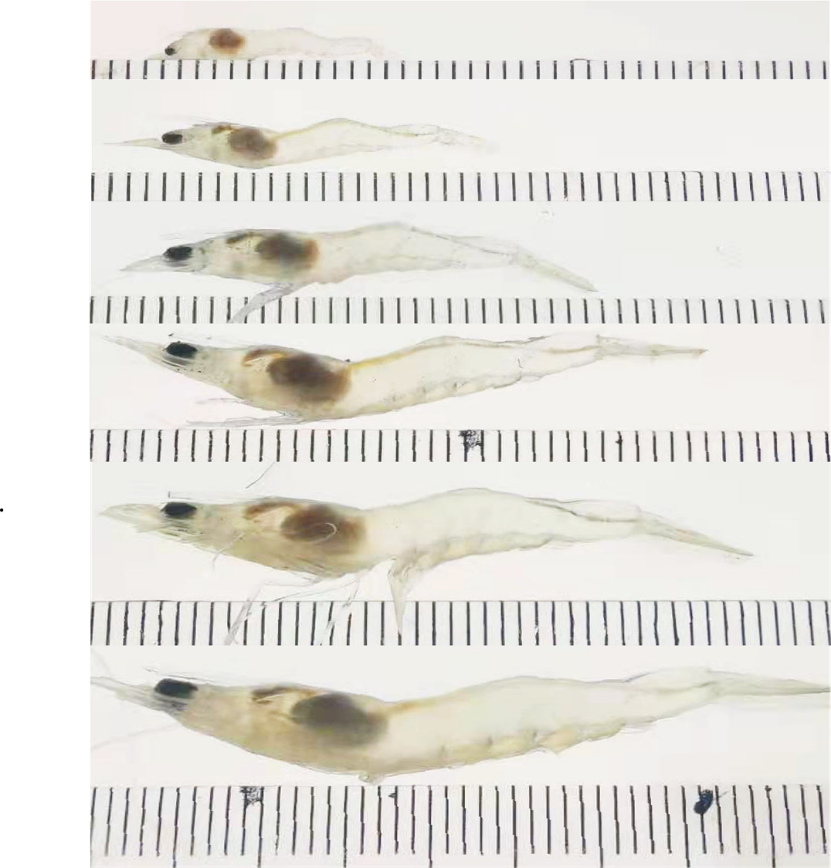


**Supplementary Figure 1.** Different growth stages of *Litopenaeus Vannamei*. Before breeding, clean the feeding tank and add disinfection effervescent tablets for 24h. The aquaculture water is adjusted to an appropriate salinity temperature of 5 parts per thousand, and the initial water temperature is 25-26℃, then increased by 2℃ every day until 30℃. Advance aeration. Air shrimp seedling even bags into the water for 1-2h, and then into the tank. Shrimp shelled eggs were fed twice a day, 5% of body weight, and water was changed once a day.


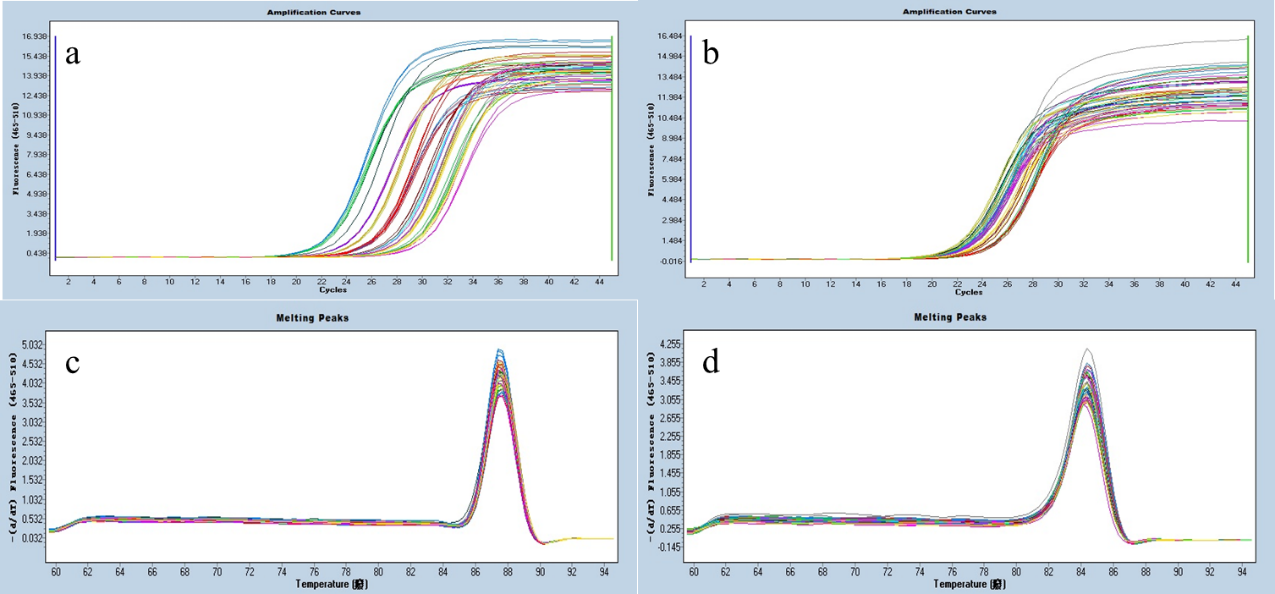


**Supplementary Figure 2.** Amplification and melting curves. a,c: SecA gene; b.d: *vp*28+mOrange gene.


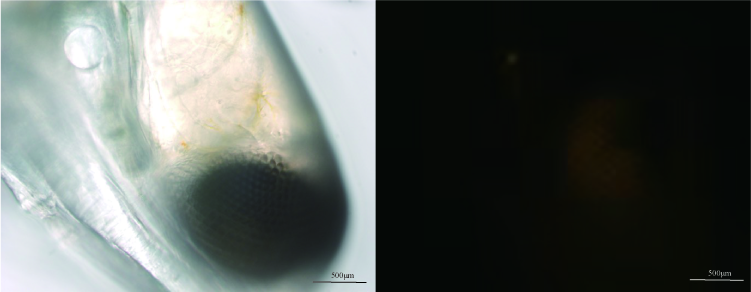


**Supplementary Figure 3.** Metabolism of VP28-mOrange fusion protein in *L. vannamei* eyes.

## Supplementary Tables

**Supplementary Table 1** PCR and overlap PCR primers in construction. The double underlined part is the restriction site; the italic part is the primer that overlaps the overlapping part of the PCR; the lowercase part is the linker sequence

| Primer | | Sequence |
| --- | --- | --- |
| KPNI-VP28-F | | GGTACCATGGATCTGAGCTTTACCCTGAGCGT |
| VP28-R | *TTCGCCTTTGCTCACactaccgccgccgccTTCGGTTTCGG* | |
| mOrange-2F | | *GCACCGAAACCGAAggcggcggcggtagtGTGAGCAAAGGC* |
| XHOI-mOrange-2R | | CTCGAGCTATTTATACAGTTCATCCATG |

**Supplementary Table 2** PCR primer sequence

| Primer | Sequence | Length |
| --- | --- | --- |
| vp28-1-F | CAGCGGCTACTTTA | 156bp |
| vp28-1-R | CCCACGGTGACTT |  |
| mOrange-1-F | AGTGGGAACGAGTG | 190bp |
| mOrange-1-R | CCATCTTCAGGGTAC |  |
| 16SRNA-F | CACACTGGGACTGAGACAC | 182bp |
| 16SRNA-R | CTGCTGGCACGGAGTTAG |  |

**Supplementary Table 3** RT-PCR primer sequence

| Primer | Sequence | Length |
| --- | --- | --- |
| vp28+mOrange-Fn | CCGTGATTGCCGTGTTTATT | 184bp |
| vp28+mOrange-Rn | TTGCCCAGGGTATCGCTAT |  |
| SecA-F | ACGACGGTCAGATTGCCGAGAT | 204bp |
| SecA-R | GCGACATTCCCTGCTGGATTAG |  |

﻿

# pRL489-vp28-mOrange plasmid sequence. The italic character is *vp*28 gene sequence, the bold character is *mOrange* gene sequence, and the underscore character in the middle is linker gene sequence.

AAACCTTGCTAGTTCTGAAAGTTCCAACGCCTCAGCACTGTTAACAGAATTTGTTCGTTGGTATTTGGCAGGTAACAGGTTTAATACTCCCACTTCTCACACTCCCACCCATCTAGACACATCCCTCGAACAGCGTATAGACAATATTGAACAACGTCTAGATAAAGTCACAACTAATAATCTAGACAATATAGATGAATTTATAGACAAGCGTATAGAAGATAATCTAGCAACACGTCTAGACAAACTTCAATCGCAACTGGAGGAACTGCGGGGAAAATCGAAAGCCCGGTAGTTCAGGCAGAAGGACAAGCTACCGGGCAAGACAGAAAGAATATAGACAATAGTATAGACAATCTAGACAAATTGGAGGCAACCCGCGATCGCACCCTCAATAAGCTAAAAATGGGTAGGCAGTCAGCCGCCGGGAAAGCCATCGACGCGTTTATCAAAGAGTTGCTTTCTTCAGGAGACAACATAAGCTGAAGTTATCAAAATTCTGTCCTTACGTCGAAAGCCTGATTTTACCGTGCAACGATTGATAAGCTTGGCTAAACTAGCACTGGCTTTCAACAGAAAGCATACGAAGAATCAATAGATATAGCCACCAATTCCACAAAATGCAGATAACGTGTAGAGTATTGGAATGCTTAATCTGTAAGGGTTATGAAGGTTAACGGCAACGGACGAGCCAAAATACTCACCTCCGACGAACTCAGGCGACTGTTTAGCGACGGATTCACCACACCGCGCGATCGCGTTTTGTTTGGCATCTGTCTATTCACCGGTTGCCGCGTTAGTGAAGCTCTAGCACTCCAAACAACGGACATTAAAGGCGAAACACTAACCTTTAGGAAGTCTACCACCAAAGGGAAACTCAAAACCCGCGTGGTTGACATCCAGCCAGGACTAGCCGCACTCATGGCTGACTATCACCCCAAACCGGGAACCCTGTTCCCTGGCATGAGGGGAGTCAGCGATAGGCTCACGCGATACGCGGCGGATAAAATCTTGCGCGATGCAGCCAAAAGAATCGGGCTAGAAGGCATCAGTACCCACAGTTTCCGCCGTACTGCCCTCAACCAAATGTCTAGCGCCGGTATCCCGTTGCGACACATTCAAGAGATATCCGGTCACAATGACCTTGGCACACTGCAACGCTATCTTGAAGTTACACCCGAACAGCGACGCAAAGCTGTATCCGTGATTGGCTTCTAATGTACGCCAACGCTGTTTAGACCCCTATGGGTGCTAAAAAAAGACGCAGCCTAAACACACGCTCTACACTTGAGGATACTTTTAAAGTATCCATCGGTTCTAGAACTCTGCACACGTTCCGGACTTTGGAAACGTTATACCTTTCCCTGTGTTGCAGAATGCTGCAATATTTCTTCGACAAGTTAACTTGTGACTGGTTTAATATTTTCTCAAATTGCCCCAAAACAACACGCCTAAATCCTTAGACGTTTCTGTGGAAACCTATTAGGTTTTTATCGCCGTTGTTTTAGTGGTAAACCCAAAGGGTTTGTATATTCTTGTATGAAGTTCGACTCTGAGGGTTAAGAAGAATGGCTCGCCGAATTTTTTACAAGTGGAAACCGATTAAAGGTTAAGGGTCAATCGGGACGATGAATATTTTCTAATTGTGACCTTCTCCATCTAATAAGCTTTCTTTGGGGTTAAGGTCGAAGAAAGTACTACGCATGATCTGCATACGATCTCTATTGCCAAAAAGCCGCGACCCTATAGGCTCTCGGTCATGCTGCACTAGTTCGTGTCGATCACTATACTGGTTGCCGCAGCATTTCACGCTAAAAAAAAATTCTTAAAAATGTCCTTCATATCTCGCCAGAGTGGCAACCTATTACAAAACGGTTGCCTACCCGACCGGCTCGATTTTCGCTGAAGTGGCACTGTGACAGTTTGAAATGGTACTTCCGCCGTGCTGCTGACATCGTTGTTAGGGTGAATTGTTCGCGGTAGATGTTGCACCGATTCATGAACACCTTGTCACCCACTTTGAATAATCGACCGTCAAATTCAGTCGCGTCAATTTGGTAAGTGTTGGGCTGTCTCTTTTTGGCTCCAGGGGCAATGCCATCAGAAAACACAACCGCGTCACCCATAACTTGATAACCGATATCAGTTTTGGTTCCAGTGAAAGCCCAAAATTCAGACGCGTCATTATTCCGAGCGTGCCGGAGTTGATTGTACTCAATTTTGGCTTGGCAAAGTTGACGGCGATTCATGCCCAGCTGCTTTTGATGTCGTCGCACTGTGCGCTTGTGAATACCCAACTCACAGCTGACAGCTTTTTGAGATGTACCATAGTGGATGAAACTTTTTGAGACGAATATCCGCGACGAACTAATGTGAAGTACACAAGGTACTTCCCCCTCTGGCGATTTAAGAGAGGATTGCCTTGTGTCCTTCACTAGCTCGTTCGGGTGTGGCGCTCCAAAAAGTTTTCTGTACTCTGGTTTAAGTTGTCTGTTGGCCGCATAGCGGCTCTTTTGTTGAAAGCTTTGTGTGACTATGCCAGTGGTCAGTGAGCGTAAATCGCTTAACACTTGGACTAAAGGCACTACTGCAACATCACCCCATCTTTTTAAATTTAGGTTGTAACAAACTTGAAACATACCGCCCAAGTAGACGGTTATCATTCCTGCTTTAATTTTGTAGCGGCGGAATGCTCCTATTTTTTTTCCATCCTGTAACCAACGGTAAACAGACTTATCACTACAATCTAAGAACGTCTGTACTACAGGCAATGGCAATGTTAAATGACCAGACCCATCCTTATCAAGCGCTCGACACAAATACCACAACCGCGCACAAGGTTCTCGACCAATGCGAGTGTGTACCCTGACCGTGTAAGTGCCAAGAATTATTTCAGTTTGTAGTTCCCTTGTAAGCAGGGTTAGTGATACATTTGTATTTAAGCTTTCTGGGCTGATCATTTGGAAATGTCTCAGTCCAGTACCTATTGAATGTTATTTGCTTAACCTGAAGCTAAATAAAACTTGTTAACTACACCCATTAATTGATAAATTCAAAGCACGTTTTTTCTGTTTGGTGTTTGGTGTGGTAACAATTCTGTGTATGTGTGTTTTATTTAGCTTCGGTTAAGTAGCATAACAACCCCCAAGCACTGAACTTTTTTTAATAGGTAATTTAAACTTTGCCTATCGGCAAAATTTTCAATCAATTGTACGCCAAAGTGTTGCATGATCAACGTTTGACTTATTTTTGTATTTACTAAATACTGAATTTCGCCGTGACGCTTTTTACAGATGGAAATTCACGGCAAAATGTTTTTTGCTAACTTTGCTATGTAAAACAAGAAACTTGGCACTCGGTTATTACTAAATAAACTGGTAAAAAATAACCATTAGAACCAAAAAGAACGAAAACCAGTACACCCTTGCCAGTTTTCAAGCTTTTGCTATGACGACTCTAATAATCGGGTTTAACACCATTCCGCTTTGAGAAAATTATCCTTGTACAGCAAGTAACAGTCAATGCTAAACCGCACCGCTACAAATCCTTAAGTTTTTCCAGTAGCGATTTACCTTCTTGGTAACGCCCGCCTTGATAGCCCAAAATTTCTTTAATCACCTTACTTTCTGAAAAACCCGCTTCCAGACAGGCTTTTACCACTTTTGCTAGGGTTTCATCTCTTGGTTCTGGGAGGGATGAAACGGGCTGTAATGCTTGTTCTGAGGTCGGTTGAGCCGTTTGGAGTGGCTGAAAACTGGTTACAGACTGTAACCGGGGCATAACCATTTTGTAACTGCTTACATCTGGTAACTGACACGGCATATCATCCACCATGCAGCGATATTTCCCCGACTTTAACCACTCCACAAGGGCAAGGTCTTTTAAGGACTTGGCGTGGCTAACTGCAAACTTACCCAGGCGTAACATCCTAAAACACTTACGGACACCGCCTTCACCCTCGATACCTAAGGTCTTGACATTATCATCTTGAGTCAGCCCAATAACAAAACGCTTGGGCTTGCGGCCGCGCCTGGCGTGTTTGATGAGCCATTCGGTTGCTATCTCGACTTCATCTCTCAGCAGTGGCAGTTCTTCAGCAATTAAAACGCTTTCTTTTCCTGCTAGTGCCTTATCCCCAGACTCACCCCGTAGCTCAATCCGGCGCTGCAATTCCTCCAGGTCAGCAGCCATGCCCGACTGTATAGCCTCAAAGTCACCACGGCGGCCAATGACATTTAACCCCGTCCACTCGTCCGGTGCAGCGTCAGCGTCATAGACTGTCACCTCACCCCCGACTTGATAAGCAAGCCATTGGGCTATGGTGCTTTTGCCAGTTCCCGTATCCCCAACTATTAAACAGTGCTTACCAGACAGAGCTTGCATCAAGTCGGTGATGATTCCCTCTGGTTCGACCGCAAGGGTGACGGCGGTAGTGTCAATGATAGCCGCGCCGTAAGTGCCAGCATAGGGCAATTGGTCGTAAACTTTGACCAAGTTGTATACAGACTGTCTACACCACTTCACCACTGTTAACGCTGTTTGCAAAGCGTAAGACGTGGCATCAAATAAAAATATGCTGGCACTAAAAGTTAATCGCCCCAATCCCCACAGTAAAAACCTGCCTAGCTGTTGACGACTAGGCAAGTGCATTTCAATCCAGTCATTTGCCATAAATCACCCCGTCTTTAAAGCCTTGCAGTTGAGCGCGACAGGTATTTAACTGTGCTTGTAACTCTGTTTGCTGGTTTTGATACCACAGACTGACGGCGGCGGCCGCCAGTCCTAAAAATAGAAACTGGCGATCGCTCATTATTGACTTACTCCCTGTTGATTAGCGTGGTAGTGAGTCATAGCCGCATTGACCGCTTCTTGGGCTTGGGGTGTTCTGCCAAGATTGGGTTTTGTAGGGTCATCGTTGGCTACGACTAAGGACGCTTGTTCGGCTATCGCTTGCGGGACACCAACTTTAGTTAACTCTGTCAAGGATACTTGGTAAAGTCGCTCGTTCATTAGCCGATTCTCCGGTACATAAAACTGTTGCTGGCAGTCCCTTCATTGGCGACGAGTTCTTCAGCCGGAGTATCAGCGATAATGTCAGCCCAGCCGGTGACATTATTATTAATAATGTTTTGTTCGGCAATTGCACCCAAGCCAGGACGCGCCGTTTCAAACTCAGAGATGACTTGCTGCTCTTTCTCGGTGAGTGGTCTATCTGTCATGATAATTATGTCCTTCATTATGTAGGCGATTCCAGTGGGTGTTTACGAGGCAGTCCACAGGAATCAGTGCGATTCACCTTTAAGGTGAATCGTCATCAAAAAATCACTCGGTAGCAACGACCCGAACCGACCAGGATTGATTTCCCGGTTCTCAGTTCGCAGGCTTTTGAGCGCGTCACCTTGACCATTGGGTAACTGCCATCAGCCGATAAGCTAAACGGGCTGTATAGCGGTAAAGCATCCCACACAGTCGGGCTGGCATCAACTTTGCAGGAATAGCTCACGTCACTCATCTCACTCGCGCCTGGGTTGGATGGCAGCGAAGGCAGATTACGACGCAGTTTTTTACTGGCACTTTTACCCGCATTAAAAACGGGTACAGTGCCATTGTTGACGGTCTGTACTTCGGTCATATACTCGGTGTACACTTAATACACTCTATACTATTACTGCCGATTAGTACATTTGTCAATCACTCTTTGCACAAGGTGTATGATATGGACTCAGGAGTACACCAAACGTCATGCCAACCAATAAAGGGAGAATAGCAGTCACTCTAGAAGCTGAAATTTACCAATGGATTGCTAACCGAGCGTCTGAGGAAGGAAGACCGTTGGCTAATCTTGCCGCTTTCTTACTCACACGAGTTGTTAAAGAACAAATGGAACAAGAAGCCAAGGACAACCAAGACAAGCAGGGGGCAGCATGAGCGAAGACAGACTAGCCAGAATAGAAGCTGCGTTAGACAGCCAAGTTGCAGTGAATGCCGACCTCCGCACATCGGTTACAGAACTCCGCGCAACCGCAGAAGCATTGTTGCAAACAGTTCAAATCCATCAGCAGAACTTTGAAATTCTTACCGCTAGGCAATTACAAACCGAAGCACGGCTTGATGAGTACCAACGTACCACTAGCGCGGCACTCGACAGAATTGGCGCGGTCTTAGACTACCTCGTTAGGCAGCAAAACGGTTGAGGTGAGGGATGAGCGATGACTATCTAGACGGATATCCCGCAAGAGGCCCTTTCGTCTTCAAGAATTCCCGTTTGACTGGCGATGCTGCTACTGAAACTAACAACTACATCGACTACGCAATTAACGCCCTCAGCTAATTTTGCTTAGTCTAGGCCCGGATGGGTAAGTGGTTTTCAGCTTAAGTGTTGGGTTCTACTTACTTCTCCGGGTCTTGCTCTATCTAAAAACATTGGTTTAACAAGGAGTATTAGGCAAATGCCAGTTACTGTCGCTGCCTCTCGCTTGGGAACCGCTGCGTTTGACCAATCACCCGTCGAACTGCGCGCTAACTATTCTCGACCTGCAGGTCGACTCTAGAGGATCTCAATGAATATTGGTTGACACGGGCGTATAAGACATGTTATACTGTTGAATAACAAGGACGGATCCGggtacc*ATGGATCTGAGCTTTACCCTGAGCGTGGTGAGCGCCATTCTGGCCATTACCGCCGTGATTGCCGTGTTTATTGTGATTTTTCGCTACCATAATACCGTGACCAAAACCATTGAAACCCATACCGGCAATATTGAAACCAATATGGATGAAAATCTGCGCATTCCCGTGACCGCCGAAGTCGGCAGCGGCTACTTTAAAATGACCGATGTCAGCTTTGATAGCGATACCCTGGGCAAAATCAAAATCCGCAACGGCAAAAGCGATGCCCAAATGAAAGAAGAAGATGCCGATCTGGTGATCACCCCCGTGGAAGGCCGCGCCCTGGAAGTCACCGTGGGCCAGAACCTGACCTTTGAAGGCACCTTCAAAGTCTGGAACAATACGAGCCGAAAAATCAATATCACCGGAATGCAGATGGTTCCCAAAATCAACCCGAGCAAAGCCTTTGTGGGCAGCTCGAACACCAGCAGCTTCACTCCCGTCAGCATCGACGAAGATGAAGTGGGCACGTTTGTTTGCGGCACCACCTTCGGCGCCCCCATCGCGGCGACAGCTGGTGGCAACTTGTTCGATATGTATGTTCACGTCACCTACAGTGGCACCGAAACCGAA*GGCGGCGGCGGTAGT**GTGAGCAAAGGCGAAGAAAATAATATGGCCATTATTAAAGAATTTATGCGCTTTAAAGTGCGCATGGAAGGCAGCGTGAATGGCCATGAATTTGAAATTGAAGGCGAAGGCGAAGGCCGCCCCTACGAAGGCTTTCAAACCGCCAAACTGAAAGTGACCAAAGGCGGCCCCCTGCCCTTTGCCTGGGATATTCTGAGCCCCCAATTTACCTACGGCAGCAAAGCCTACGTGAAACATCCCGCCGATATCCCTGACTATTTTAAATTGAGCTTCCCTGAGGGATTTAAGTGGGAACGAGTGATGAACTTTGAGGATGGTGGGGTCGTGACGGTGACCCAAGATAGTAGCCTACAGGATGGTGAATTTATCTATAAAGTTAAACTGAGAGGCACCAATTTTCCTAGTGATGGGCCCGTGATGCAGAAGAAGACCATGGGCTGGGAAGCTAGTAGTGAACGTATGTACCCTGAAGATGGCGCCTTGAAGGGTGAAATTAAAATGCGCCTGAAACTGAAAGATGGGGGCCATTACACCAGCGAAGTGAAAACCACCTACAAAGCCAAAAAACCCGTGCAACTACCCGGCGCCTACATTGTCGGCATTAAACTGGATATCACTAGCCATAATGAAGATTACACCATCGTTGAGCAGTATGAACGCGCCGAAGGCCGCCATAGCACCGGCGGCATGGATGAACTGTATAAATAG**ctcgagCAATTACTCAAAATTTCTACCAGATGATAATGGAAAGCTTACAGACAGGAACCATTAGCTCTGATAGTGATTACATTCAATTTCCTAAGGTTGATGTATATCCCAAAGTGTACTCAAAAAATGTACCAACCTGTATGACTGCTGAGTCCGCAAGTACGACAGAATGGCTAGCAATACAAGGGCTACCAATGGTTCTTAGTTGGATTATTGGTACTAATGAAAAAAAAGCACAGATGGAACTCTATAATGAAATTGCGACAGAATATGGTCATGATATATCTAAAATAGATCATTGTATGACTTATATTTGTTCTGTTGATGATGATGCACAAAAGGCGCAAGATGTTTGTCGGGAGTTTCTGAAAAATTGGTATGACTCATATGTAAATGCGACCAATATCTTTAATGATAGCAATCAAACTCGTGGTTATGATTATCATAAAGGTCAATGGCGTGATTTTGTTTTACAAGGACATACAAACACCAATCGACGTGTTGATTATAGCAATGGTATTAACCCTGTAGGCACTCCTGAGCAGTGTATTGAAATCATTCAACGTGATATTGATGCAACGGGTATTACAAACATTACATGCGGATTTGAAGCTAATGGAACTGAAGATGAAATAATTGCTTCCATGCGACGCTTTATGACACAAGTCGCTCCTTTCTTAAAAGAACCTAAATAAATTACTTATTTGATACTAGAGATAATAAGGAACAAGTTATGAAATTTGGATTATTTTTTCTAAACTTTCAGAAAGATGGAATAACATCTGAAGAAACGTTGGATAATATGGTAAAGACTGTCACGTTAATTGATTCAACTAAATATCATTTTAATACTGCCTTTGTTAATGAACATCACTTTTCAAAAAATGGTATTGTTGGAGCACCTATTACCGCAGCTGGTTTTTTATTAGGGTTAACAAATAAATTACATATTGGTTCATTAAATCAAGTAATTACCACCCATCACCCTGTACGTGTAGCAGAAGAAGCCAGTTTATTAGATCAAATGTCAGAGGGACGCTTCATTCTTGGTTTTAGTGACTGCGAAAGTGATTTCGAAATGGAATTTTTTAGACGTCATATCTCATCAAGGCAACAACAATTTGAAGCATGCTATGAAATAATTAATGACGCATTAACTACAGGTTATTGTCATCCCCAAAACGACTTTTATGATTTTCCAAAGGTTTCAATTAATCCACACTGTTACAGTGAGAATGGACCTAAGCAATATGTATCCGCTACATCAAAAGAAGTCGTCATGTGGGCAGCGAAAAAGGCACTGCCTTTAACATTTAAGTGGGAGGATAATTTAGAAACCAAAGAACGCTATGCAATTCTATATAATAAAACAGCACAACAATATGGTATTGATATTTCGGATGTTGATCATCAATTAACTGTAATTGCGAACTTAAATGCTGATAGAAGTACGGCTCAAGAAGAAGTGAGAGAATACTTAAAAGACTATATCACTGAAACTTACCCTCAAATGGACAGAGATGAAAAAATTAACTGCATTATTGAAGAGAATGCAGTTGGGTCTCATGATGACTATTATGAATCGACAAAATTAGCAGTGGAAAAAACAGGGTCTAAAAATATTTTATTATCCTTTGAATCAATGTCCGATATTAAAGATGTAAAAGATATTATTGATATGTTGAACCAAAAAATCGAAATGAATTTACCATAATAAAATTAAAGGCAATTTCTATATTAGATTGCCTTTTTGGGGATCCTTTTTGATAATCTCATGACCAAAATCCCTTAACGTGAGTTTTCGTTCCACTGAGCGTCAGACCCCGTAGAAAAGATCAAAGGATCTTCTTGAGATCCTTTTTTTCTGCGCGTAATCTGCTGCTTGCAAACAAAAAAACCACCGCTACCAGCGGTGGTTTGTTTGCCGGATCAAGAGCTACCAACTCTTTTTCCGAAGGTAACTGGCTTCAGCAGAGCGCAGATACCAAATACTGTCCTTCTAGTGTAGCCGTAGTTAGGCCACCACTTCAAGAACTCTGTAGCACCGCCTACATACCTCGCTCTGCTAATCCTGTTACCAGTGGCTGCTGCCAGTGGCGATAAGTCGTGTCTTACCGGGTTGGACTCAAGACGATAGTTACCGGATAAGGCGCAGCGGTCGGGCTGAACGGGGGGTTCGTGCACACAGCCCAGCTTGGAGCGAACGACCTACACCGAACTGAGATACCTACAGCGTGAGCTATGAGAAAGCGCCACGCTTCCCGAAGGGAGAAAGGCGGACAGGTATCCGGTAAGCGGCAGGGTCGGAACAGGAGAGCGCACGAGGGAGCTTCCAGGGGGAAACGCCTGGTATCTTTATAGTCCTGTCGGGTTTCGCCACCTCTGACTTGAGCGTCGATTTTTGTGATGCTCGTCAGGGGGGCGGAGCCTATGGAAAAACGCCAGCAACGCGGCCTTTTTACGGTTCCTGGCCTTTTGCTGGCCTTTTGCTCACATGTTCTTTCCTGCGTTATCCCCTGATTCTGTGGATAACCGTATTACCGCCTTTGAGTGAGCTGATACCGCTCGCCGCAGCCGAACGACCGAGCGCAGCGAGTCAGTGAGCGAGGAAGCGGAAGAGCGCCTGATGCGGTATTTTCTCCTTACGCATCTGTGCGGTATTTCACACCGCATATGGTGCACTCTCAGTACAATCTGCTCTGATGCCGCATAGTTAAGCCAGTATACACTCCGCTATCGCTACGTGACTGGGTCATGGCTGCGCCCCGACACCCGCCAACACCCGCTGACGCGCCCTGACGGGCTTGTCTGCTCCCGGCATCCGCTTACAGACAAGCTGTGACCGTCTCCGGGAGCTGCATGTGTCAGAGGTTTTCACCGTCATCACCGAAACGCGCGAGGCAGCTGCGGTAAAGCTCATCAGCGTGGTCGTGAAGCGATTCACAGATGTCTGCCTGTTCATCCGCGTCCAGCTCGTTGAGTTTCTCCAGAAGCGTTAATGTCTGGCTTCTGATAAAGCGGGCCTGCCACCATACCCACGCCGAAACAAGCGCTCATGAGCCCGAAGTGGCGAGCCCGATCTTCCCCATCGGTGATGTCGGCGATATAGGCGCCAGCAACCGCACCTGTGGCGCCGGTGATGCCCGAAGAACTCCAGCATGAGATCCCCGCGCTGGAGGATCATCCAGCCGGCGTCCCGGAAAACGATTCCGAAGCCCAACCTTTCATAGAAGGCGGCGGTGGAATCGAAATCTCGTGATGGCAGGTTGGGCGTCGCTTGGTCGGTCATTTCGAACCCCAGAGTCCCGCTCAGAAGAACTCGTCAAGAAGGCGATAGAAGGCGATGCGCTGCGAATCGGGAGCGGCGATACCGTAAAGCACGAGGAAGCGGTCAGCCCATTCGCCGCCAAGCTCTTCAGCAATATCACGGGTAGCCAACGCTATGTCCTGATAGCGGTCCGCCACACCCAGCCGGCCACAGTCGATGAATCCAGAAAAGCGGCCATTTTCCACCATGATATTCGGCAAGCAGGCATCGCCATGGGTCACGACGAGATCATCGCCGTCGGGCATGCGCGCCTTGAGCCTGGCGAACAGTTCGGCTGGCGCGAGCCCCTGATGCTCTTCGTCCAGATCATCCTGATCGACAAGACCGGCTTCCATCCGAGTACGTGCTCGCTCGATGCGATGTTTCGCTTGGTGGTCGAATGGGCAGGTAGCCGGATCAAGCGTATGCAGCCGCCGCATTGCATCAGCCATGATGGATACTTTCTCGGCAGGAGCAAGGTGAGATGACAGGAGATCCTGCCCCGGCACTTCGCCCAATAGCAGCCAGTCCCTTCCCGCTTCAGTGACAACGTCGAGCACAGCTGCGCAAGGAACGCCCGTCGTGGCCAGCCACGATAGCCGCGCTGCCTCGTCCTGCAGTTCATTCAGGGCACCGGACAGGTCGGTCTTGACAAAAAGAACCGGGCGCCCCTGCGCTGACAGCCGGAACACGGCGGCATCAGAGCAGCCGATTGTCTGTTGTGCCCAGTCATAGCCGAATAGCCTCTCCACCCAAGCGGCCGGAGAACCTGCGTGCAATCCATCTTGTTCAATCATGCGAAACGATCCTCATCCTGTCTCTTGATCAGATCTTGATCCCCTGCGCCATCAGATCCTTGGCGGCAAGAAAGCCATCCAGTTTACTTTGCAGGGCTTCCCAACCTTACCAGAGGGCGCCCCAGCTGGCAATTCCGGTTCGCTTGCTGTCCATAAAACCGCCCAGTCTAGCTATCGCCATGTAAGCCCACTGCAAGCTACCTGCTTTCTCTTTGCGCTTGCGTTTTCCCTTGTCCAGATAGCCCAGTAGCTGACATTCATCCGGGGTCAGCACCGTTTCTGCGGACTGGCTTTCTACGTGTTCCGCTTCCTTTAGCAGCCCTTGCGCCCTGAGTGCTTGCGGCAGCGTGAAGCTTTCTCTGAGCTGTAACAGCCTGACCGCAACAAACGAGAGGATCGAGACCATCCGCTCCAGATTATCCGGCTCCTCCATGCGTTGCCTCTCGGCTCCTGCTCCGGTTTTCCATGCCTTATGGAACTCCTCGATCCGCCAGCGATGGGTATAAATGTCGATGACGCGCAAGGCTTGGGCTAGCGACTCGACCGGTTCGCTGGTCAGCAACAACCATTTCAACGGGGTCTCACCCTTGGGCGGGTTAATCTCCTCGGCCAGCACCGCGTTGAGCGTGATATTCCCCTGTTTTAGCGTGATGCGCCCACTGCGCATA
